# Thromboembolism after COVID-19 vaccine in patients with preexisting thrombocytopenia

**DOI:** 10.1038/s41419-021-04058-z

**Published:** 2021-08-03

**Authors:** Alessandro Mauriello, Manuel Scimeca, Ivano Amelio, Renato Massoud, Antonio Novelli, Francesca Di Lorenzo, Susanna Finocchiaro, Carolina Cimino, Rossana Telesca, Marcello Chiocchi, Qiang Sun, Ying Wang, Yufang Shi, Giuseppe Novelli, Gerry Melino

**Affiliations:** 1 Department of Experimental Medicine, TOR, University of Rome Tor Vergata, 00133 Rome, Italy; 2 School of Life Sciences, University of Nottingham, Nottingham, UK; 3 Laboratory of Medical Genetics, Translational Cytogenomics Research Unit, Bambino Gesù Children Hospital, IRCCS, Rome, Italy; 4 Department of Biomedicine and Prevention, University of Rome Tor Vergata, 00133 Rome, Italy; 5 Laboratory of Cell Engineering, Institute of Biotechnology; Research Unit of Cell Death Mechanism, Chinese Academy of Medical Science, 2020RU009, 20 Dongda Street, Beijing, 100071 China; 6 Institute of Nutrition and Health Sciences, Shanghai Institutes for Biological Sciences, Chinese Academy of Sciences, Shanghai, China; 7 The Third Affiliated Hospital of Soochow University, Changzhou, China; 8 The Child Health Institute of New Jersey, Rutgers-Robert Wood Johnson Medical School, New Brunswick, NJ USA; 9 Institutes for Translational Medicine, Soochow University, Suzhou, China; 10 IRCCS Neuromed, Pozzilli, IS Italy; 11 Department of Pharmacology, School of Medicine, University of Nevada, Reno, NV 89557 USA; 12 DZNE German Center for Neurodegenerative Diseases, Bonn, Germany

**Keywords:** Diseases, Medical research

## Abstract

While vaccination is the single most effective intervention to drastically reduce severe disease and death following SARS-CoV-2 infection, as shown in UK and Israel, some serious concerns have been raised for an unusual adverse drug reaction (ADR), including vaccine-induced immune thrombotic thrombocytopenia (VITT) with concurrent low platelets as well as capillary leak syndrome. In fact, the overall safety of the vaccine is highlighted by the low frequency of ADR considering that in UK, by the early June, 40 million first doses and 29 million second doses have been injected; nonetheless, 390 thrombotic events, including 71 fatal events have been reported. Interestingly, the cases reported low platelet counts with the presence of anti-platelet factor-4 (PF4) antibodies, indicating an abnormal clotting reaction. Here, out of three referred cases, we report a post-vaccine clinical case of fatal thrombosis with postmortem examination and whole exome sequencing (WES) analysis, whose pathogenesis appeared associated to a preexisting condition of thrombocytopenia due to myelodysplasia.

## Introduction

Therapeutic approaches to cure COVID-19 are still under development, despite the recent significant progress in the design of monoclonal antibodies [1, 2], inhibitors of proteases [3–5], and the identification of novel targets, such as the HETC E Ligase, Itch [6, 7]. The vaccination against the infection of SARS-CoV-2 has been proven so far as the most effective intervention to limit damage to human health and to prevent expansion of the economical and societal implications associated to the pandemic [8–10]. While governments and health institutions have deployed major resources to promptly reach heard immunity by vaccination, reports of rare thrombosis events have threated the regular course of the programmes. Following immunization with adenovirus-vector COVID-19 vaccines ChAdOx1 nCOV-19 (AstraZeneca) and Ad26.COV2·S (Johnson&Johnson/Janssen), cerebral venous thromboses have been observed with specific cases resulted in fatal outcome [11–13]. Little scientific literature is currently available and most of the investigated cases appeared associated to a clinical picture of moderate-to-severe thrombocytopenia and thrombotic complications beginning 10–15 days after vaccination, resembling severe heparin-induced thrombocytopenia (HIT) [14]. HIT is well-characterized as a prothrombotic condition triggered by antibodies against platelets, that recognize the molecular complex generated by interaction of cationic platelet factor-4 (PF4) and anionic heparin [14]. Frequently, patients with post COVID-19 vaccination thrombosis displayed high level of anti-PF4 antibodies in absence of heparin administration, thus leading to the definition of vaccine-induced immune thrombotic thrombocytopenia [13].

Here out of three referred cases, we report a case of fatal thromboembolism following administration of the first dose of ChAdOx1 nCOV-19 (AstraZeneca). We conducted an in-depth postmortem analysis, which revealed a bone marrow focal megakaryocyte hyperplasia associated with morphological dysplastic changes. The patient also displayed peripheral thrombocytopenia, with undetectable level of serum anti-PF4 antibodies. WES revealed no significant genetic alterations in genes associated to thrombocytopathies, complementopathies, and platelets disfunction diseases. Thus, the underlying myelodysplasia appeared causative of a vaccine-independent preexisting thrombocytopenia that predisposes to severe adverse drug reaction to the COVID-19 immunization.

## Results and discussion

Starting from day +1 after receiving the vaccine ChAdOx1 nCOV-19 (Oxford-Astrazeneca), a 48-year-old Caucasian woman reported progressive headache, back pain, moderate right lower limb pain, and disseminated ecchymosis that required hospitalization on day +18 for 7 days (S. Eugenio Hospital, Rome).

The clinical history included allergy to penicillin and an episode of thrombocytopenia in 2016. At the physical examinations, the patient appeared apyretic, with good hemodynamic compensation, absence of abnormal sounds at respiratory auscultation, soft abdomen and normal neurological objectivity, with a blood pressure of 214/125 mmHg. Laboratory tests showed a D-dimer value >10000 ng/mL and thrombocytopenia lower than 32000 platelets/μL. A pulmonary angio-CT showed thrombo-embolic filling defects affecting the pulmonary artery, in absence of any densitometric changes in the parenchyma. Venous echo-color doppler of the lower limbs showed absence of previous and/or concurrent deep venous obstructions. Low molecular weight heparin was administered.

**Fig. 1:**
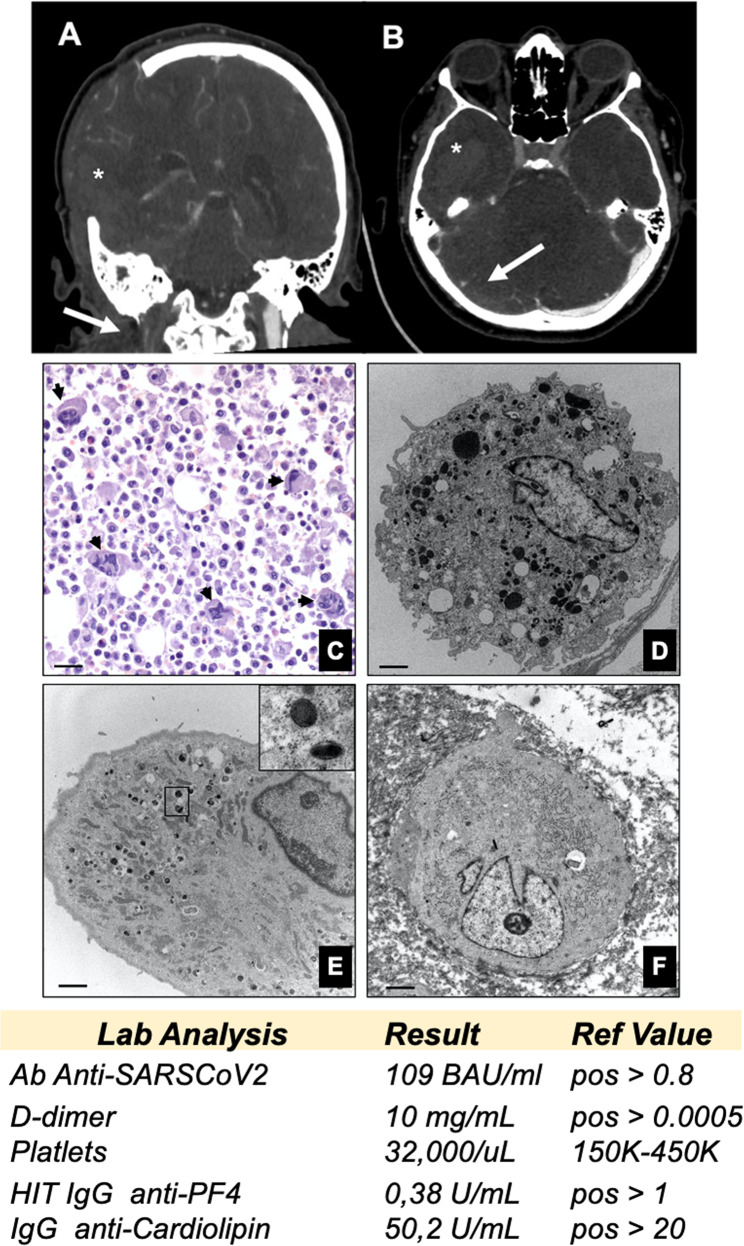
Thrombosis and preexisting myelodysplasia. Computed tomography angiography (CTA); **A** coronal view shows right internal jugular vein thrombosis (arrow), **B** axial view shows right transverse sinus thrombosis (arrow). A massive hemorrhagic focus is observed on right hemisphere (*). **C** Hematoxylin and eosin stain shows numerous degranulated megakaryocytes (arrows) (scale bar 100 µm). **D** Normal megakaryocyte characterized by large and abundant cytoplasm with more than 50 mature platelet granules (scale bar 5 µm). **E** Electron micrograph displays a megakaryocyte with few mature platelet granules (square) and numerous mitochondria (scale bar 3 µm). **F** A completely degranulated megakaryocyte (scale bar 5 µm). Table reports laboratory test results (full results in Supplementary Table 2).

On day +22, the patient required a transfer to the neurology intensive care unit to improve her management, as a significative intensification of headache, confusion, nausea, and vomiting emerged. The new therapeutic strategy included anti-hypertensive, and oral double (dabigatran 110 mg/die + rivaroxaban 30 mg/die) anti-coagulants in addition to i.v. methylprednisolone. In the following two days a drastic worsening of patient’s neurological symptoms including altered consciousness, strength deficit in the left side and right gaze deviation. An emergency skull CT documented massive right temporo-occipital intraparenchymal hemorrhage, extending to the ventricular system associated to midline shift; CT angiography investigating intra- and extra-cranial circulation showed thrombotic phenomena of the sigmoid transverse sinus and of the right internal jugular vein (Fig. 1A). The administration of Dabigatran antagonists and a decompressive craniectomy (day +23) was urgently performed. The patient remained comatose in intensive care for about 2 weeks, till day +16 after surgery (or day +39), when brain death was diagnosed.

**Fig. 2:**
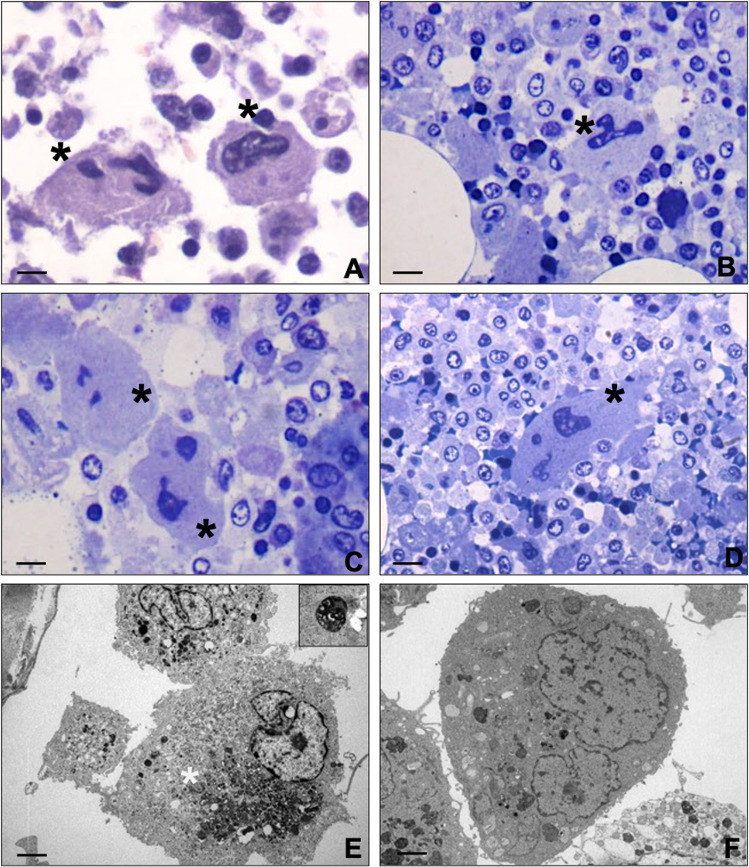
Light and transmission electron microscopy of bone marrow. **A** Haematoxylin and eosin stain shows two large size degranulated megakaryocytes (asterisks) (scale bar 20 µm). **B** Toluidine blue stain displays a degranulated megakaryocytes with a large and segmented nucleus (asterisks) (scale bar 20 µm). **C, D** Degranulated megakaryocytes with fragmented nuclei (asterisks) (scale bar 10 µm). **E** Electron micrograph shows a megakaryocyte with rare immature/degenerated platelet granules. The cytoplasm included large amount of degenerating electrodense material (asterisk). The square highlights high magnification of an immature platelet granule (scale bar 5 µm). **F** A megakaryocyte with no/rare platelet granules (scale bar 5 µm).

**Fig. 3:**
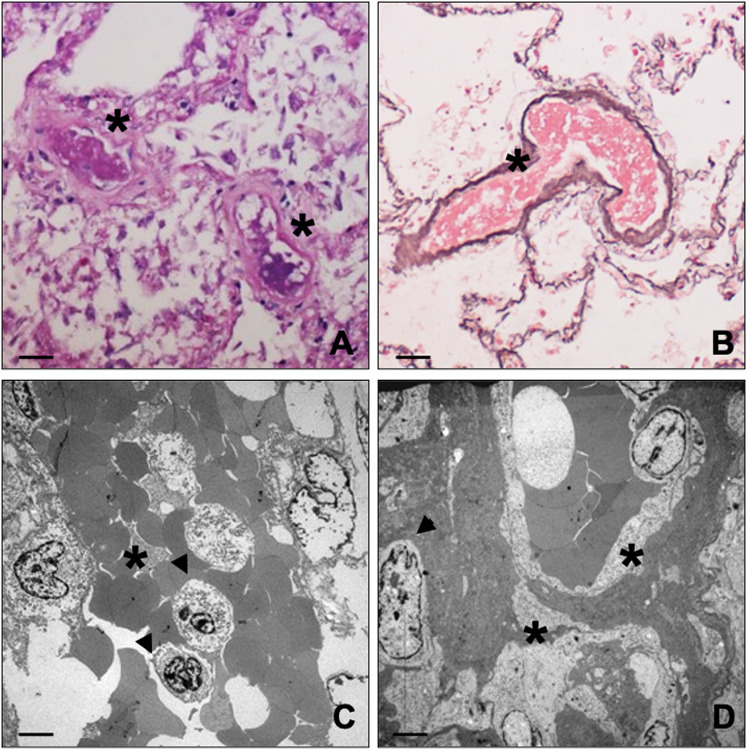
Histological analysis of lung thrombi. **A**–**D** Histological and electron microscopy analysis of lung tissues. Both PAS (**A**) and silver (**B**) staining show vascular thrombi (asterisks) in lung capillaries (scale bar 100 µm). **C** Electron micrograph shows vascular thrombi characterized by microvascular fibrin deposition (asterisks). The thrombi include degenerated monocytes (arrows) (scale bar 5 µm). **D** Image displays vascular thrombi with microvascular fibrin deposition (asterisks) and well-preserved pericytes (arrows) (scale bar 5 µm).

Autoptic examination revealed a massive cerebral hemorrhage complicated by a purulent abscess involving the right fronto-temporo-parietal lobes, the nucleus of the right base, with midline shift and wedging of the cerebellar tonsils and an internal and external haemocephalus (Fig. 1B and Supplementary Fig. 1). Bilateral confluent foci of bronchopneumonia associated to a right apical pulmonary infarction of both lungs were also observed (Supplementary Fig. 2A, B). Postmortem analysis of bone marrow, including hematoxylin and eosin stain, immunohistochemistry, and transmission electron microscopy (TEM), showed focal megakaryocyte hyperplasia associated with morphological dysplastic changes. Abnormalities included non-lobulated or hypo-lobulated megakaryocytes, increase of cytoplasm, and separate or dark pycnotic nuclei (Fig. 1C–F and Fig. 2). Normal megakaryocytes (30–70 μm of diameter), characterized by large and hyperploid nuclei, accounted only 20% of the total population (Fig. 1B) and displayed more than 50 platelet granules (65,56 ± 8.67) (Fig. 1D). Conversely, the largest majority of megakaryocytes (~70%) displayed a significant impairment in the platelet granule formation (Fig. 1E, F), with degranulated cytoplasm and rare immature platelet granules (11.45 ± 4.14) (Fig. 1E). Five to ten percent of megakaryocytes were characterized by a complete impairment of platelet granules formation (Fig. 1F). TEM analysis of lung tissues further confirmed the frequent occurrence of vascular septic thrombi characterized by microvascular fibrin deposition (Fig. 3). Degenerated inflammatory cells, such as monocytes, were observed in several organized thrombi; vessels displayed no pericytes alteration. Signs of long standing petechiae were noted on the skin; hematoxylin–eosin staining reported a sclerosis of the dermis associated to nonspecific vasculitis infiltrated by CD3-positive T lymphocytes (Supplementary Fig. 3). All together, the ultrastructural data suggest the occurrence of a thrombocytopenia related to the impairment of megakaryocytes differentiation. Nevertheless, a paradoxical effect was observed in the lung with diffuse pulmonary thrombosis.

We also performed a WES genetic analysis on the patient’s PBMC. We studied more in details 64 genes involved in thrombocytopathies, complementopathies, and several platelets disfunction diseases [15, 16] and detected a total of 585 genetic variants. Introducing a cutoff at MAF 0.01, we selected 11 rare putative variants (1,88%): 1 out of these is a missense variant, 10 out of the total are intronic variants (Supplementary Table 1). According to the American College of Medical Genetics guidelines, these variants can be classified as benign and/or of unknown significance; also, the *SERPINF2* missense variant emerged as benign and tolerated in all in silico prediction tools.

While the patient displayed several autoimmune autoantibodies (Supplementary Table 2), we failed to detect anti-PF4 antibodies, in contrast with most of the reported cases [11–14, 17], but in keeping with a similar case of ChAdOx1 nCOV-19 vaccine (Astrazeneca/Oxford) administration [18]. This suggests that in addition the immune thrombotic thrombocytopenia associated with antibodies that recognize PF4 and activate platelets through their Fcγ receptors, leading to autoimmune HIT [19, 20], other causes of thrombocytopenia, such as megakaryocyte dysplasia, could lead to potential life-threatening ADR. The underlying molecular events are not clear, even though platelets express ACE2, which can be triggered by SARS-CoV-2 binding to enhance thrombosis [21]; hence suggesting a stoichiometric relationship leading to the thrombotic event. Conversely, the vaccine administration has been associated to events of anti-PF4/polyanion antibodies production [22], but not in our specific case. We also detected the presence of NETosis, in keeping with its driving role in HIT [23], but only at a late stage and in the brain tissue, whilst the lung was depleted of neutrophils. Therefore, in keeping with the highly complex pattern of immune response to SARS-CoV-2, a sensible recommendation seems to be to avoid vaccination in thrombocytopenic patients and/or myelodysplastic patients or at least keep them under very strict control (https://www.gov.uk/government/publications/coronavirus-covid-19-vaccine-adverse-reactions/ coronavirus-vaccine-summary-of-yellow-card-reporting).

## Material and methods

### Histology

Several samples of heart (left and right ventricles, septum, coronary tree), lungs (at least one for each lobe), liver, spleen, kidneys, bone marrow, brain, cerebellum, transverse sinus of dura mater, and skin (long standing petechiae) were fixed in buffered formalin and paraffin embedded. Serial sections for each sample were stained with hematoxylin and eosin (H&E). Also, PAS, Reticulum and Grocott silver stains were performed.

### Immunohistochemistry

Immunohistochemical analyses were performed on 3-μm-thick paraffin sections. Briefly, EDTA citrate pH 7.8 buffer was used for the antigen retrieval. Sections were then incubated with the following pre-diluted primary antibodies: CD3 (clone Cd 2GV6, Rabbit Monoclonal, Ventana, Roche), CD20 (clone L26, mouse monoclonal), CD68 (clone KP-1, mouse monoclonal), CD117 (clone EP10, rabbit monoclonal), CD34 (clone QBEnd/10, mouse monoclonal), CD138 (clone B-A38, mouse monoclonal), kappa (rabbit polyclonal), lambda (rabbit polyclonal), and Myeloperoxidase (rabbit polyclonal) as previously described (https://www.gov.uk/government/publications/coronavirus-covid-19-vaccine-adverse-reactions/ coronavirus-vaccine-summary-of-yellow-card-reporting). Reactions were revealed by the HRP-DAB Detection Kit (UCS Diagnostic, Rome, Italy).

### Transmission electron microscopy (TEM)

One cubic millimeter of tissue from each collected specimen was fixed in 4% paraformaldehyde and post-fixed in 2% osmium tetroxide. After washing with 0.1 M phosphate buffer, the sample was dehydrated by a series of incubations in 30, 50, and 70% ethanol. Dehydration was continued by incubation steps in 95% ethanol, absolute ethanol, and propylene oxide; then, samples were embedded in EPON (Agar Scientific, Stansted Essex CM24 8GF United Kingdom). Eighty micrometers ultra-thin sections were mounted on copper grids and observed with MORGAGNI 268 D transmission electron microscope (FEI, Hillsboro, Oregon, USA).

### Whole exome sequencing and data pre-processing

Genomic DNA was extracted from peripheral blood samples using standard procedures and the Qiagen blood DNA mini Kit (Qiagen, Hilden, Germany). Library pre-paration and whole exome capture were performed by using the Twist Human Core Exome Kit (Twist Bioscience, South San Francisco, CA, USA) according to the manufacture’s protocol and sequenced on the Illu- mina NovaSeq 6000 platform. The BaseSpace pipeline (Illumina, Inc., San Diego, CA, USA) and the TGex software (LifeMap Sciences, Inc., Alameda, CA, USA) were used for the variant calling and annotating variants, respectively. Sequencing data were aligned to the hg19 human reference genome. A minimum depth coverage of 30X was considered suitable for analysis, based on the guidelines of the American College of Medical Genetics and Genomics. All variants were examined for coverage and Qscore (minimum threshold of 30) and visualized by the Integrative Genome Viewer.

## Supplementary information

Supp Figures 1-3

Supp Table 1-2
