# Supplementary material for: Thromboembolism after COVID-19 vaccine in patients with preexisting thrombocytopenia
Source: Cell Death Dis. 2021 Aug 3;12(8):762. doi: 10.1038/s41419-021-04058-z (PMC8328816; doi:10.1038/s41419-021-04058-z)
Supplement: Supplementary file 1 — Supp Figures 1-3 [file 41419_2021_4058_MOESM1_ESM.pptx]

## Slide 1
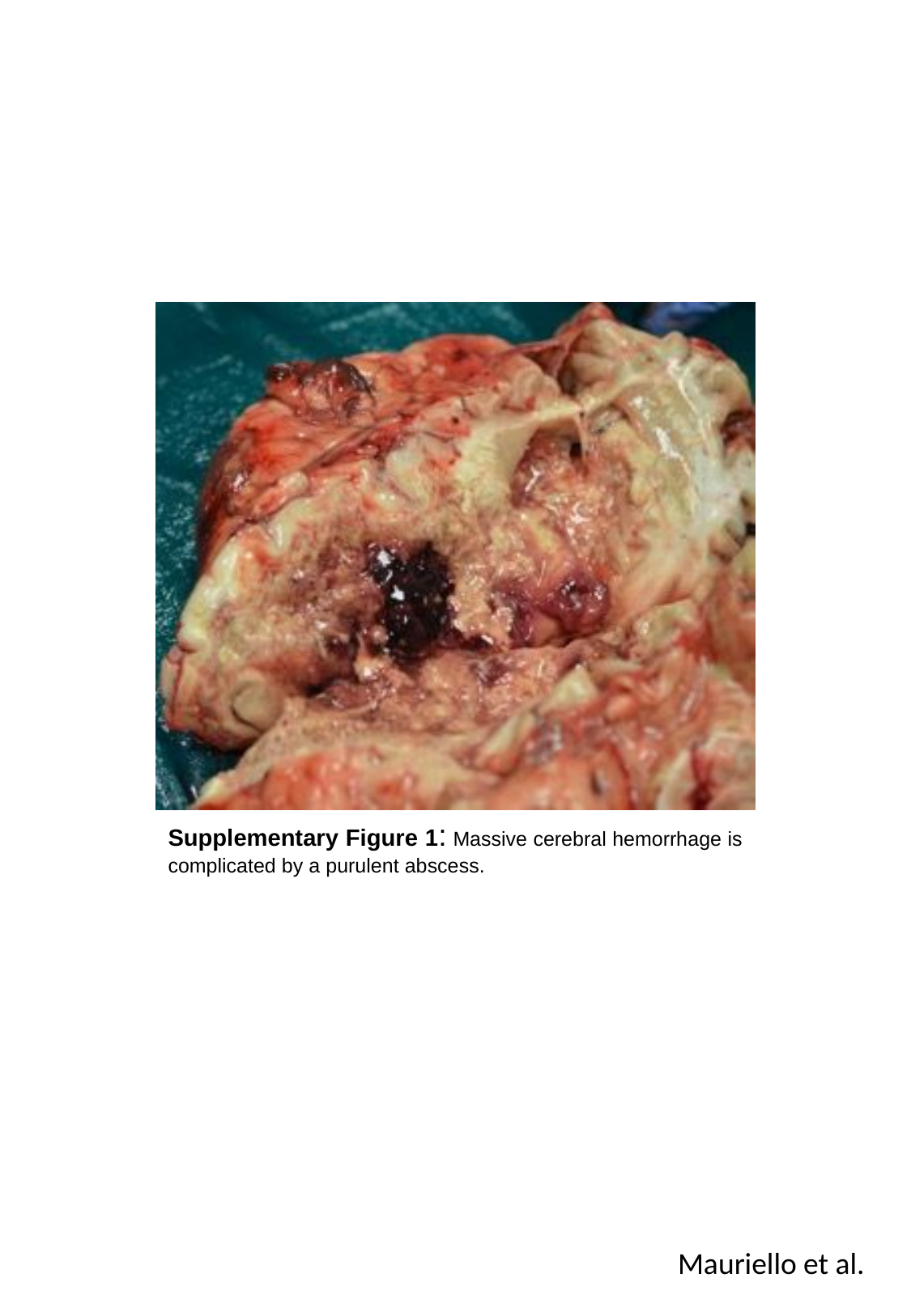

Supplementary Figure 1: Massive cerebral hemorrhage is complicated by a purulent abscess.
Mauriello et al.

## Slide 2
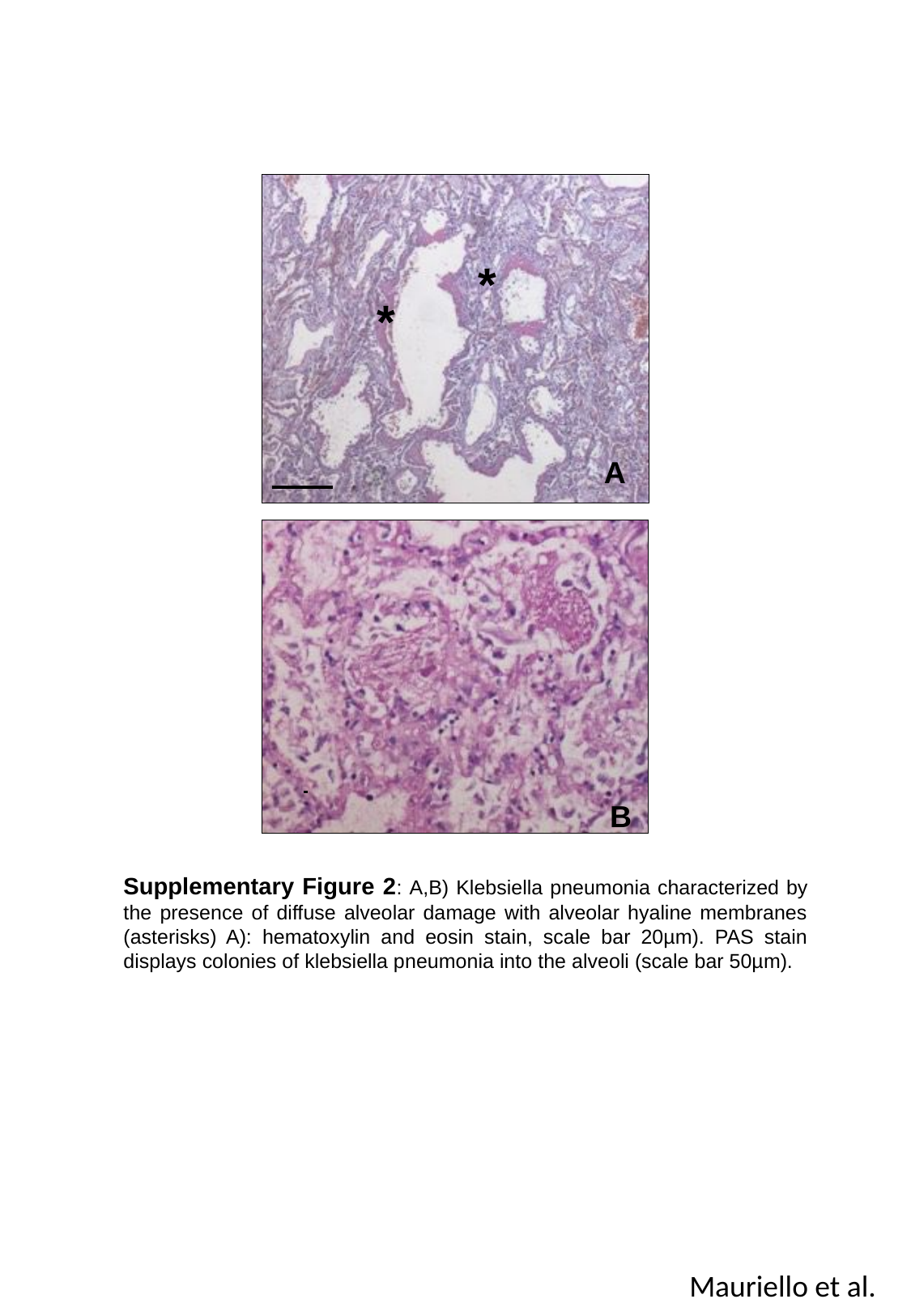

*
*
A
B
Supplementary Figure 2: A,B) Klebsiella pneumonia characterized by the presence of diffuse alveolar damage with alveolar hyaline membranes (asterisks) A): hematoxylin and eosin stain, scale bar 20µm). PAS stain displays colonies of klebsiella pneumonia into the alveoli (scale bar 50µm).
Mauriello et al.

## Slide 3
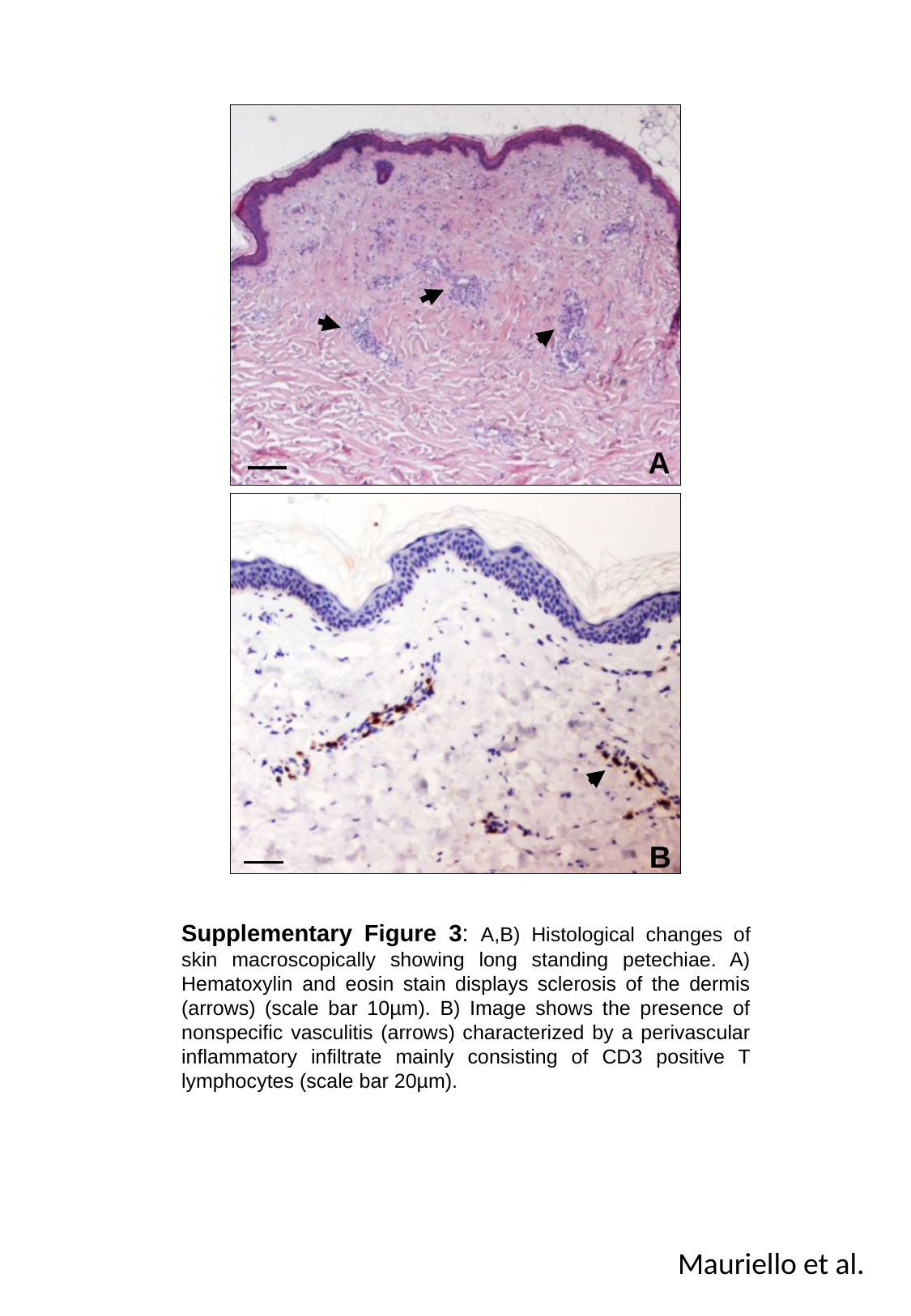

A
B
Supplementary Figure 3: A,B) Histological changes of skin macroscopically showing long standing petechiae. A) Hematoxylin and eosin stain displays sclerosis of the dermis (arrows) (scale bar 10µm). B) Image shows the presence of nonspecific vasculitis (arrows) characterized by a perivascular inflammatory infiltrate mainly consisting of CD3 positive T lymphocytes (scale bar 20µm).
Mauriello et al.
