# Supplementary material for: Thromboembolism after COVID-19 vaccine in patients with preexisting thrombocytopenia
Source: Cell Death Dis. 2021 Aug 3;12(8):762. doi: 10.1038/s41419-021-04058-z (PMC8328816; doi:10.1038/s41419-021-04058-z)
Supplement: Supplementary file 2 — Supp Table 1-2 [file 41419_2021_4058_MOESM2_ESM.pdf]

Gene	HGNC ID	Location	HGVSc	HGVSp	Type	RefSeq	dbSNP	Max AF	MT	PP2	SIFT	ClinVar	VarSome
HIVEP1	4920	6:12130955	c.6210-45G>A	/	Intronic	NM_002114.3	/	0,0000000000	/	/	/	/	/
KLKB1	6371	4:187171374	c.599-22_599-20delTCT	/	Intronic	NM_000892.4	rs773453149	0,0000079537	/	/	/	N.A.	VoUS
SERPINF2	9075	17:1651905	c.729C>G	p.Asn243Lys	Missense	NM_000934.3	rs763091299	0,0000079647	/	Benign	Tolerated	N.A.	VoUS
CFB	1037	6:31918896	c.1856-25_1856-24insA	/	Intronic	NM_001710.5	rs749107211	0,0000162479	/	/	/	N.A.	VoUS
VWF	12726	12:6125422	c.5312-24G>A	/	Intronic	NM_000552.4	rs375741263	0,0000636902	/	/	/	N.A.	Likely Benign
GFI1B	4237	9:135862915	c.238+109C>T	/	Intronic	NM_004188.6	rs141328455	0,0022944551	/	/	/	N.A.	Benign
CFB	1037	6:31914669	c.299-115T>A	/	Intronic	NM_001710.5	rs4151668	0,0029687799	/	/	/	N.A.	VoUS
RUNX1	10471	21:36261949	c.-2540C>G	/	Intronic	NM_001001890.2	rs529894328	0,0050632911	/	/	/	N.A.	Benign
RUNX1	10471	21:36261949	c.98-2556C>G	/	Intronic	NM_001754.4	rs529894328	0,0050632911	/	/	/	N.A.	Benign
SERPINF2	9075	17:1646560	c.-5+358T>G	/	Intronic	NM_000934.3	rs77513353	0,0065319908	/	/	/	N.A.	VoUS
SERPINF2	9075	17:1648564	c.102+57G>T	/	Intronic	NM_000934.3	rs75500393	0,0094280327	/	/	/	N.A.	VoUS

Supplementary Table 1: Variants identified in patient by Whole Exome Sequencing (WES) analysis with a MAF ≤ 0.01

*Legend of Table: **HGNC ID:** Hugo Gene Nomenclature Committee Identifier; **HGVSc:** Human Genome Variation Society coding sequence name; **HGVSp:** Human Genome Variation Society protein sequence name; **RefSeq**: Reference Sequence; **dbSNP**: Single Nucleotide Polymorphism Database; **Max AF**: Maximum Allelic Frequency; **MT**: Mutation Taster; **PP2**: PolyPhen2 - Polymorphism Phenotyping v2; **SIFT**: Sorting Intolerant From Tolerant; **HIVEP1:** HIVEP Zinc Finger 1; **KLKB1:** Kallikrein B1; **SERPINF2:** Serpin Family F member 2; **CFB:** Complement Factor B; **VWF:** Von Willebrand Factor; **GFI1B:** Growth Factor Independent 1 transcriptional repressor**; RUNX1:** RUNX family transcription factor 1.*

Test	Result	Reference Value
**FLC K,** Free Light Chains Ratio	**81 mg/L**	**6,70-22,40**
**FLC L,** Free Light Chains	**56,4 mg/L**	**8,30-27**
IgM Beta-2 Glycoprotein 1 Antibodies	2,8 U/mL	NEGATIVE
ENA7, Extractable Nuclear Antigen Antibodies	17,6 CU	NEGATIVE
MPO, Myeloperoxidase	3,8 CU	NEGATIVE
IgG Beta-2 Glycoprotein 1 Antibodies	12,3 U/mL	NEGATIVE
PR3, Proteinase 3 antineutrophil cytoplasmic	< 2,3 CU	NEGATIVE
**IgG anti-Cardiolipin**	**50,2 U/mL**	**POSITIVE >20**
dsDNA	12,9 IU/mL	NEGATIVE
**IgM anti-Cardiolipin**	1,7 U/mL	NEGATIVE
**HIT IgG (anti PF4)**	**0,38 U/mL**	**≤ 1,00 U/mL**
**Ab anti SARS-COV2 Spike S**	**108,5**	**POSITIVE > 0,8**

Supplementary Table 2: Laboratory Test results.
